# P-242. Disparities and inequities in the burden of pneumococcal disease in US adults

**DOI:** 10.1093/ofid/ofae631.446

**Published:** 2025-01-29

**Authors:** Salini Mohanty, Kelly D Johnson, Sheba Nellore, Lindsay McNamee, Hanane Khoury, Laura De Benedetti, Elmira Flem, Kristen A Feemster, Nicole Cossrow

**Affiliations:** Merck & Co., Inc, Rahway, New Jersey; Merck & Co., Inc, Rahway, New Jersey; Certara, Montreal, Quebec, Canada; Certara, Montreal, Quebec, Canada; Certara, Montreal, Quebec, Canada; Certara, Montreal, Quebec, Canada; Merck Sharp & Dohme LLC, North Wales, Pennsylvania; Merck & Co., Inc., Rahway, New Jersey; Merck & Co, Inc., Kenilworth, New Jersey

## Abstract

**Background:**

Understanding the impact of social determinants of health on pneumococcal disease (PD) is important. The purpose of this study was to conduct a targeted literature review (TLR) of the clinical and economic burden of PD in US adults with a focus on examining disparities and inequities by race, geography, urbanicity, income, education, and employment.

**Methods:**

Original research studies reporting burden of PD in US adults published between 2012 and January 2024 were identified through a US focused TLR in Pubmed (vial Medline) and a review of surveillance data from the CDC website. The original search was supplemented with targeted searches via Google Scholar in areas for which data were not identified via Pubmed or the CDC website.

**Results:**

Of 4,133 identified publications, 16 studies were identified: 10 described disparities by race, 7 by geography, 2 by urbanicity, 3 by income, 1 by education and 1 by employment. Black adults had the highest incidence and longest length of stay due to IPD and bacteremic community acquired pneumonia (CAP) and highest mortality rates from CAP compared to other racial groups across all adult age groups; Black adults living in poverty experienced a 2.1 times higher rate of PD compared to white adults. Pneumococcal disease also varied by geography and urbanicity. Lower urbanicity displayed higher mortality rates for PD. Incidence of all-cause CAP was higher in early retirees 18–64 years old and their adult dependents compared to their employed counterparts and their adult dependents. PD health care expenditures mirror the patterns observed for disease burden. Vaccination rates were lower in Black adults compared to white adults and in adults who resided in rural areas, were less educated and had a lower income.

**Conclusion:**

This TLR confirms that disparities and inequities in the burden of PD among US adults exist with higher disease burden and lower pneumococcal vaccination in Black adults, those living in rural areas and with lower education and income. There is a paucity of studies examining inequities and social disparities of health in PD. Additional studies are needed to examine disparities and inequities in PD according to race, geography, urbanicity, income, education, and employment.

**Disclosures:**

**Salini Mohanty, DrPH, MPH**, Merck & Co., Inc.: Employee|Merck & Co., Inc.: Stocks/Bonds (Public Company) **Kelly D. Johnson, PhD**, Merck & Co., Inc.: Employee|Merck & Co., Inc.: Stocks/Bonds (Public Company) **Sheba Nellore, MBA**, Merck: Advisor/Consultant **Lindsay McNamee, BSc**, Merck & Co Inc.: Advisor/Consultant **Hanane Khoury, PhD**, Merck: Advisor/Consultant **Laura De Benedetti, BSc**, Merck & Co, Inc: Advisor/Consultant **Elmira Flem, MD, PhD**, Merck: Stocks/Bonds (Private Company) **Kristen A. Feemster, MD, MPH, MSHPR, FAAP**, Merck & Co., Inc., Rahway, NJ, USA: Grant/Research Support|Merck & Co., Inc., Rahway, NJ, USA: Stocks/Bonds (Public Company) **Nicole Cossrow, PhD**, Merck: Stocks/Bonds (Public Company)

